# Properties and Expression of Na^+^/K^+^-ATPase α-Subunit Isoforms in the Brain of the Swamp Eel, *Monopterus albus*, Which Has Unusually High Brain Ammonia Tolerance

**DOI:** 10.1371/journal.pone.0084298

**Published:** 2013-12-31

**Authors:** Xiu L. Chen, Nicklaus L. J. E. Wee, Kum C. Hiong, Jasmine L. Y. Ong, You R. Chng, Biyun Ching, Wai P. Wong, Shit F. Chew, Yuen K. Ip

**Affiliations:** 1 Department of Biological Sciences, National University of Singapore, Singapore, Republic of Singapore; 2 Natural Sciences and Science Education, National Institute of Education, Nanyang Technological University, Singapore, Republic of Singapore; National Institutes of Health/NICHD, United States of America

## Abstract

The swamp eel, *Monopterus albus*, can survive in high concentrations of ammonia (>75 mmol l^−1^) and accumulate ammonia to high concentrations in its brain (∼4.5 µmol g^−1^). Na^+^/K^+^-ATPase (Nka) is an essential transporter in brain cells, and since NH_4_^+^ can substitute for K^+^ to activate Nka, we hypothesized that the brain of *M. albus* expressed multiple forms of Nka α-subunits, some of which might have high K^+^ specificity. Thus, this study aimed to clone and sequence the *nka α*-subunits from the brain of *M. albus*, and to determine the effects of ammonia exposure on their mRNA expression and overall protein abundance. The effectiveness of NH_4_^+^ to activate brain Nka from *M. albus* and *Mus musculus* was also examined by comparing their Na^+^/K^+^-ATPase and Na^+^/NH_4_^+^-ATPase activities over a range of K^+^/NH_4_^+^ concentrations. The full length cDNA coding sequences of three *nkaα* (*nkaα1*, *nkaα3a* and *nkaα3b*) were identified in the brain of *M. albus*, but *nkaα2* expression was undetectable. Exposure to 50 mmol l^−1^ NH_4_Cl for 1 day or 6 days resulted in significant decreases in the mRNA expression of *nkaα1*, *nkaα3a* and *nkaα3b*. The overall Nka protein abundance also decreased significantly after 6 days of ammonia exposure. For *M. albus*, brain Na^+^/NH_4_^+^-ATPase activities were significantly lower than the Na^+^/K^+^-ATPase activities assayed at various NH_4_^+^/K^+^ concentrations. Furthermore, the effectiveness of NH_4_^+^ to activate Nka from the brain of *M. albus* was significantly lower than that from the brain of *M. musculus*, which is ammonia-sensitive. Hence, the (1) lack of *nkaα*2 expression, (2) high K^+^ specificity of K^+^ binding sites of Nkaα1, Nkaα3a and Nkaα3b, and (3) down-regulation of mRNA expression of all three *nkaα* isoforms and the overall Nka protein abundance in response to ammonia exposure might be some of the contributing factors to the high brain ammonia tolerance in *M. albus*.

## Introduction

Ammonia plays a crucial role in the maintenance of nitrogen homeostasis in almost all living organisms; but it is toxic if allowed to accumulate in the body. High concentration of ammonia affects the central nervous system, resulting in several neurological abnormalities [1] characterized by hyperactivity, convulsions, coma and eventually death [2]. Mammals, including humans, develop encephalopathy when brain ammonia content reaches 1–3 µmol g^−1^ [1], and ammonia remains as the leading candidate in the pathogenesis of hepatic encephalopathy in acute liver failure. Hepatic encephalopathy, if left untreated, can lead to hepatic coma and death. Several classical theories have been proposed to address the pathological consequences of increased ammonia concentration and the consequential changes in nitrogen metabolism in mammalian brains. These include glutamatergic dysfunction, glutamine accumulation leading to astrocyte swelling, and/or activation of N-methyl-D-aspartate (NMDA)-type glutamate receptors leading to ammonia-induced membrane depolarization [3], [4]. Excessive activation of NMDA-type glutamate receptors is neurotoxic, resulting in oxidative stress and subsequent degeneration and death of neurons [5]–[7]. Recent findings have pointed to an important role of glutamine-mediated oxidative/nitrosative stress [8], [9] and/or mitochondrial permeability transition [10] in the pathogenesis of cerebral ammonia toxicity.

Fishes are generally more tolerant to high internal ammonia concentrations than terrestrial vertebrates [11], but they are not exempted from the deleterious effects of high concentrations of ammonia on various cellular processes [12]–[14]. Fully aquatic fishes keep body ammonia levels low by excreting excess ammonia, mainly as NH_3_, through their gills [15]. However, some fishes dwelling in habitats with low oxygen tension have acquired the ability to breathe air, and air-breathing sometimes leads to degenerate gills with reduced functions [16]. Air-breathing fishes can be exposed to environmental ammonia when stranded in puddles of water during dry season, during a stay inside a burrow, or after agricultural fertilization. Under these conditions, accumulation of endogenous and exogenous ammonia would occur, resulting in high concentrations of ammonia in the blood and various organs. Therefore, some air-breathing fishes have developed mechanisms to defend against ammonia toxicity at the branchial/epithelial surfaces through active ammonia excretion, lowering of the external pH, reducing ammonia permeability or ammonia volatilization [12]–[14]. In others, defence against ammonia toxicity can also take place at the cellular level by detoxifying ammonia to other nitrogenous compounds (e.g. glutamine or urea) or developing high cell/tissue ammonia tolerance [12]–[14].

The swamp eel, *Monopterus albus* (Zuiew, 1793), is an anguilliform bony fish, belonging to the family Synbranchidae, order Synbranchiformes, and class Actinopterygii. It is an obligate air-breather with degenerate gills which have been reduced to an opercular skin-fold [16]. Its natural habitat includes muddy ponds, swamps, canals, and rice fields [17], where it burrows in moist earth for long periods during drought [18]. It may also encounter high concentrations of environmental ammonia (∼90 mmol l^−1^) [19] in rice fields during agricultural fertilization. Notably, the 48-h, 72-h and 96-h median lethal concentrations (LC_50_) of total ammonia for *M. albus* are 209.9 mmol l^−1^, 198.7 mmol l^−1^ and 193.2 mmol l^−1^, respectively [20], which are much higher than those for other fishes, many of which would succumb to <5 mmol l^−1^ NH_4_Cl. The LC_50_ for *M. albus* are even higher than those for some other tropical fishes known to have high environmental ammonia tolerance [21]. For instance, the 96-h LC_50_ of total ammonia for the giant mudskipper *Periophthalmodon schlosseri*, and the Boddart’s goggle-eyed mudskipper, *Boleophthalmus boddaerti*, are 115 mmol l^−1^ and 13.8 mmol l^−1^, respectively [22]. Furthermore, *M. albus* can tolerate extremely high levels of ammonia in its organs, especially the brain, during emersion or exposure to environmental ammonia [20], [23], [24]. After 144 h of exposure to 75 mmol l^−1^ NH_4_Cl at pH 7.0, ammonia concentration in the muscle, liver, brain and gut of *M. albus* reach 11.5, 15.2, 6.5 and 7.5 µmol g^−1^, respectively [20]. More intriguingly, after an intraperitoneal injection of a sublethal dose of ammonium acetate, the brain ammonia concentration transiently reaches 11.2 µmol g^−1^ [25]. Thus, unlike mammals and other fish species, the brain of *M. albus* must possess mechanisms that would help avoid the deleterious effects of ammonia, such as a disruption of ion transport, energy metabolism and other cellular processes [13].

Recently, Ip et al. [26] obtained the full cDNA coding sequence of * N* *a^+^*:* K* *^+^:2Cl^−^ cotransporter 1b* (*nkcc1b*) from the brain of *M. albus*, and reported that *M. albus* was able to down-regulate the mRNA and protein expression of *nkcc1b*/Nkcc1b in the brain, which could be one of the contributing factors to its extraordinarily high brain ammonia tolerance, when confronted with high concentrations of environmental ammonia (50 mmol l^−1^). Since Na^+^/K^+^-ATPase (Nka/NKA) is ubiquitously expressed in all cell types [27], and since NH_4_^+^ can substitute for K^+^ to activate not only NKCC1b but also NKA [28], it is logical to hypothesize that high brain ammonia tolerance in *M. albus* would involve Nka. To achieve high brain ammonia tolerance, it is imperative for Nka from the brain of *M. albus* to differentiate effectively K^+^ from NH_4_^+^, so that intracellular K^+^ homeostasis and a stable resting membrane potential can be maintained in brain cells. Furthermore, since it has been established that ammonia intoxication can lead to an increase in NKA activity resulting in ATP depletion and related deleterious consequences in mammalian brains [29], it is also logical to hypothesize that *M. albus* could down-regulate the mRNA and/or protein expression of Nka α-subunit isoforms in its brain when confronted with high brain ammonia concentrations. Of note, two different types of abbreviations were adopted in this report because the standard abbreviations of genes/proteins of fishes (http://zfin.org/cgi-bin/webdriver?MIval=aa-ZDB_home.apg) are different from those of frogs and human/non-human primates (http://www.genenames.org). Specifically, for fishes, gene symbols are italicized, all in lower case, and protein designations are the same as the gene symbol, but not italicized with the first letter in upper case. The advantage and appropriateness of using two types of genes symbols is that it would allow immediate interpretation of the affiliation between the abbreviation and fish or human/non-human primates.

NKA is a member of the P-type ATPases, responsible for the active transport of 3 Na^+^ out of and 2 K^+^ into the cell, fuelled by the hydrolysis of ATP. It is essential for cell functions which include maintaining osmotic balance and membrane potential, and driving the secondary active transport of molecules such as glucose and amino acids [30]. NKA contains 2 major subunits, α and β, and functions as a αβ heterodimer. The α-subunit is a large (110–120 kDa) protein that contains all the functional sites and is responsible for the catalytic functioning of the enzyme. Four isoforms of the *NKA α*-subunit (*α1*, *α2*, *α3*, *α4*) have been identified in mammals [27]. The *NKAα1* isoform is found in nearly all tissues, but the other isoforms are more limited in expression [31]. Since three isoforms, *NKAα1*, *NKAα2* and *NKAα3*, have been identified in mammalian brain [27], [32], this study aimed to clone the full cDNA sequences of various *nka α*-subunit isoforms from the brain of *M. albus*, and to examine whether the *M. albus* brain expressed all three isoforms. This study also aimed to determine the effects of ammonia exposure (50 mmol l^−1^ NH_4_Cl) on mRNA expression of various *nka α*-subunit isoforms, in order to test the hypothesis that ammonia would lead to down-regulation of their expression in the brain of *M. albus*. The effect of ammonia exposure on the protein abundance of Nka in the brain of *M. albus* was also examined through immunoblotting using commercially available anti-NKA antibodies. In addition, efforts were made to evaluate the differences between the effectiveness of NH_4_^+^ and K^+^ to activate Nka from the brain of *M. albus* kept in freshwater or exposed to ammonia. Finally, an attempt was made to determine whether there was any difference between the effectiveness of NH_4_^+^, in substitution of K^+^, to activate Nka from the brain of *M. albus* and that from the brain of the mouse, *Mus musculus*.

## Materials and Methods

### Ethics Statement

Approval to undertake this study was obtained from the Institutional Animal Care and Use Committee of the National University of Singapore (IACUC 021/10, protocol for *M. albus*; C11/09, protocol for *M. musculus*).

### Animals

Specimens of *M. albus* (150–250 g) were purchased from a local fish distributor in Singapore. Fish were maintained in plastic tanks in freshwater at 25°C under a 12 h: 12 h dark: light regime. No aeration was provided because *M. albus* is an obligate air-breather. No attempt was made to separate the sexes. Fish were acclimated to laboratory conditions for at least 1 week before experimentation. Food was withheld during the experimental period. Specimens of *M. musculus* were obtained and maintained by the Animal Holding Unit, National University of Singapore.

### Experimental Conditions and Collection of Samples

Control fish (total *N* = 18; *N* = 5 each for ammonia assay, Nka assay and molecular work, and *N* = 3 for Western blot) were immersed in 25 volumes (v/w) of freshwater in plastic tanks with free access to air. Fish subjected to ammonia exposure were immersed in freshwater containing 50 mmol l^−1^ NH_4_Cl (pH 7), for either 1 day or 6 days (total *N* = 18 for each group). Control fish and fish exposed to ammonia were killed with an overdose of neutralized MS-222 (0.2%) followed with a strong blow to the head. The whole brain from an individual fish was quickly excised within 2 min, frozen in liquid nitrogen and stored at −80°C until further analysis. For the measurement of Nka activity, the entire brain was suspended in 1 ml of solution containing 100 mmol 1^−1^ imidazole-HCl (pH 7.2), 300 mmol 1^−1^ sucrose, 20 mmol 1^−1^ ethylenediamine tetraacetic acid (EDTA) following the method of Zaugg [33], frozen in liquid nitrogen and stored at −80°C until further analysis.

Mice (*N* = 5) were euthanized using carbon dioxide gas and regarded as dead when there was no observable respiratory activity and no reaction to mechanical stimulation. The whole brain was quickly excised within 2 min and suspended in 1 ml of Zaugg’s solution [33], frozen in liquid nitrogen and stored at −80°C until the assay of NKA activity.

### Determination of Ammonia Concentrations in the Brain

The frozen brain samples of *M. albus* were weighed, ground to a powder in liquid nitrogen, and homogenized three times in 5 volumes (v/w) of ice-cold 6% perchloric acid at 24,000 rpm for 20 s each using an Ultra-Turrax homogenizer (Ika-werk, Staufen, Germany) with intervals of 10 s between each homogenization. The homogenate was centrifuged at 10,000×g at 4°C for 30 min to obtain the supernatant. The pH of the supernatant obtained was adjusted to between 6.0 and 6.5 with 2 mol l^−1^ KHCO_3_, and the ammonia concentration was determined according to the method of Bergmeyer and Beutler [34]. Results were expressed as µmol g^−1^ wet mass tissue.

### Determination of Nka/NKA Activity

Frozen brain samples of both *M. musculus* and *M. albus* were thawed on ice and homogenized for 2 s at 7,000 rpm using an Ultra-Turrax homogenizer. The homogenate was then centrifuged at 2000×g for 7 min at 4°C to obtain the pellet. The pellet was re-suspended in 1 ml of homogenizing buffer containing 100 mmol l^−1^ imidazole-HCl (pH 7.2), 300 mmol l^−1^ sucrose, and 1 g l^−1^ of sodium deoxycholate (without EDTA, which interfered with the subsequent phosphate analysis), and homogenized twice at 13,500 rpm for 10 s each with an interval of 10 s. The homogenized sample was centrifuged for 6 min at 2,000×g and 4°C. The supernatant obtained was assayed for Nka/NKA activity on the same day. Brain samples were pre-incubated at 25°C for 10 min in the presence of 30 mmol l^−1^ imidazole-HCl buffer (pH 7.2) and 100 mmol l^−1^ NaCl, 20 mmol l^−1^ KCl and 5 mmol l^−1^ MgCl_2_, in the presence or absence of 3 mmol l^−1^ ouabain. The reaction was subsequently initiated by the addition of 0.05 ml of 3.5 mmol l^−1^ ATP (pH 7.0), incubated at 25°C and the reaction terminated by the addition of 0.05 ml of ice-cold 100% trichloroacetic acid. The Nka/NKA activity was calculated as a difference of activities assayed in the presence and absence of ouabain.

The reaction mixture was centrifuged at 12,000×g for 2 min at 4°C. The amount of inorganic phosphate (P_i_) released from ATP during the incubation period represented the activity of Nka/NKA. An aliquot (0.4 ml) of the supernatant was diluted with 4 volumes of 100 mmol l^−1^ sodium acetate for P_i_ assay. To this diluted aliquot, 0.2 ml of 1% ascorbic acid and 0.2 ml of 1% ammonium molybdate in 0.025 mol l^−1^ H_2_SO_4_ were added. Absorbance was determined at 700 nm using a UV160 UV-VIS spectrophotometer (Shimadzu, Kyoto, Japan), and the P_i_ concentration calculated with reference to a standard made from K_2_HPO_4_ and assayed in the presence of trichloroacetic acid and sodium acetate. The protein content of the sample was determined by the method of Bradford [35]. Bovine gamma globulin dissolved in 25% glycerol was used as a standard. The activity of Nka is expressed as µmol P_i_ released min^−1^ mg^−1^ protein. To evaluate if there were changes in the affinity of Nka/NKA to its substrates (i.e. K^+^ or NH_4_^+^), enzyme activities were also determined at various sub-saturating substrate concentrations (1, 2.5, 5, 10 or 20 mmol l^−1^) of KCl or NH_4_Cl. The effectiveness of substitution by NH_4_^+^ is expressed as the ratio of Na^+^/NH_4_^+^-ATPase activity to Na^+^/K^+^-ATPase activity at various K^+^ or NH_4_^+^ concentrations.

### Total RNA Extraction and cDNA Synthesis

Total RNA was extracted from brain samples of *M. albus* using Tri Reagent™ (Sigma-Aldrich Co., St. Louis, MO, USA) and further purified using the RNeasy Plus Mini Kit (Qiagen GmbH, Hilden, Germany). After extraction, RNA was quantified spectrophotometrically using a Hellma TrayCell (Hellma GmbH & Co. KG, Müllheim, Germany) and checked electrophoretically to verify the RNA integrity. Total RNA (1 µg) isolated from the brain of *M. albus* was reverse transcribed into first strand cDNA using oligo (dT)_18_ primers and the RevertAid™ First Strand cDNA synthesis kit (Fermentas International Inc., Burlington, ON, Canada) following the manufacturer’s protocol.

### Polymerase Chain Reaction (PCR) and Cloning

Partial *nka* sequences were obtained using the primers (Forward: 5′-CAC TTC ATC CAC ATC ATC AC-3′; Reverse: 5′-ATG GCA GGG AAC CAT GTC-3′) designed from the highly conserved regions based on multiple alignments of the *nkaα1*, *nkaα2* and *nkaα3* sequences from various animal and fish species available in Genbank (http://www.ncbi.nlm.nih.gov/Genbank). PCR was carried out in a Bio-Rad Peltier thermal cycler (Bio-Rad Laboratories, Hercules, CA) using Dreamtaq™ DNA polymerase (Fermentas International Inc.) under the following cycling conditions: 95°C for 3 min, followed by 35 cycles of 95°C for 30 s, 55°C for 30 s, 72°C for 2 min and a final cycle of extension at 72°C for 10 min. PCR products were separated by electrophoresis in 1% agarose gel. Bands of the expected sizes (∼3000 bp) were excised and purified from the gel using FavorPrep™ Gel Purification Mini Kit (Favorgen Biotech Corp., Ping-Tung, Taiwan) according to the manufacturer’s protocol. Purified PCR products were ligated into pGEM-T easy vector (Promega Corporation, Madison, WI, USA), transformed into JM109 *Escherichia coli* competent cells and plated onto Luria-Bertani (LB) agar with ampicillin, IPTG and X-gal. Colony-PCR was performed on selected white colonies. Colonies with insert of estimated size were grown overnight in LB/ampicillin broth in a shaking incubator (37°C, 250 rpm). Plasmid extraction was performed using AxyPrep™ Plasmid Miniprep Kit (Axygen Biosciences, Union City, CA, USA). Multiple clones of each fragment were sequenced bidirectionally by cycle sequencing using BigDye® Terminator v3.1 Cycle Sequencing Kit (Applied Biosystems Inc., Foster City, CA, USA), and subsequently purified by ethanol/sodium acetate precipitation. Purified products were automatically sequenced using the 3130XL Genetic Analyzer (Applied Biosystems Inc.). The fragments were verified to be *nka α*-subunit isoforms from GenBank database. Cloning results obtained from the sequencing of the extracted plasmid inserts indicated the presence of three isoforms of *nka α*-subunits (*nkaα1* and *nkaα3a* and *nkaα3b*).

### Rapid Amplification of cDNA Ends (RACE)

Total RNA (1 µg) isolated from the brain of *M. albus* was reverse transcribed into 5′-RACE-Ready cDNA and 3′-RACE-ready cDNA using the SMARTer™ RACE cDNA Amplification kit (Clontech Laboratories, Mountain View, CA, USA). RACE-PCR was performed using Advantage® 2 PCR kit (Clontech Laboratories) to generate the 5′ and 3′ cDNA fragments. RACE primers (Table 1) were designed based on the partial cDNA sequences obtained for all three isoforms of *nka α*-subunits. RACE-PCR cycling conditions were: 25 cycles of 94°C for 30 s, 65°C for 30 s and 72°C for 4 min. RACE-PCR products were separated using gel electrophoresis, purified and sequenced.

**Table 1:** Primer sequences for RACE and quantitative (q) RT-PCR.

Gene	Primer type		Primer sequence (5′-3′)
*nkaα1*	RACE-PCR	5′-RACE	GTC TCT CTT CAG GAT GGG AAT GTT GC
		3′-RACE	CTT CCT GGC TGA GCA GAG CAA CA
	q RT-PCR	Forward	GTT GCT TCT CCT ACT ACC AAG AG
		Reverse	ATC ACC AAC CAC TAC ATC CT
*nkaα3a*	RACE-PCR	5′-RACE	GCG CTT AAG GAT GGG CAA AGA TTC
		3′-RACE	TCA CGA AAC TGA GGA TGA AAA TGA CAA T
	q RT-PCR	Forward	AGT GGG TCA AGT TCT GTC GT
		Reverse	GTG GGC TCG TTC TCT GTG
*nkaα3b*	RACE-PCR	5′-RACE	GAT CTT GTT CTT CAT GCC CTG CT
		3′-RACE	GTG ATG TGG TGA GTG GTG ATG ATG
	q RT-PCR	Forward	TGT CTT GTG GCT CAG TCA GGA
		Reverse	GCG GTT GTC ATT AGG ATC TTC TG

Primers used for RACE and q RT-PCR of *Na^+^/K^+^-ATPase* (*nka*) α-subunit isoforms from the brain of *Monopterus albus*.

### Deduced Amino Acid Sequences and Phylogenetic Analysis

The partial fragments of *nkaα1*, *nkaα3a* and *nkaα3b* obtained were aligned using BioEdit version 7.0.9 [36] to obtain their full-length nucleotide coding sequences, which were subsequently translated into deduced amino acid sequences using ExPASy Proteomic server (http://web.expasy.org/translate/). The deduced amino acid sequences were aligned and compared with selected Nka/NKA α-subunit isoforms from various animal species using BioEdit to confirm the identity of the Nka α-subunit isoforms from *M. albus*. Transmembrane domains were identified using MEMSAT3 and MEMSAT-SVM provided by PSIPRED protein structure prediction server (http://bioinf.cs.ucl.ac.uk/psipred/) [37]. Multiple sequence alignments using the deduced amino acid sequences from selected species (*Oreochromis mossambicus*, *Xenopus laevis*, *Rattus norvegicus* and *Homo sapiens*) were also performed using ClustalW (http://www.genome.jp/tools/clustalw/).

Amino acid sequences of Nka/NKA α-subunit isoforms from other animals were obtained from Genbank of UniProtKB/TrEMBL with the following accession numbers: *Acanthopagrus schlegelii* Nkaα [ABR10300.1], *Anabas testudineus* Nkaα1a [AFK29492.1], *A. testudineus* Nkaα1b [AFK29493.1], *A. testudineus* Nkaα1c [AFK29494.1], *Carassius auratus* Nkaα3 [BAB60722.1], *Catostomus commersonii* Nkaα [CAA41483.1], *Danio rerio* Nkaα1 [NP_571761.1], *D. rerio* Nkaα2 [NP_571758.1], *D. rerio* Nkaα2a [AAI63629.1], *D. rerio* Nkaα3a [NP_571759.2], *D. rerio* Nkaα3b [NP_571760.2], *Electrophorus electricus* Nkaα [AAK27722.1], *Fundulus heteroclitus* Nkaα1 [AAL18002.1], *F. heteroclitus* Nkaα2 [AAL18003.1], *Oncorhynchus masou* Nkaα1a [BAJ13363.1], *O. masou* Nkaα1b [BAJ13362.1], *Oncorhynchus mykiss* Nkaα1a [AAQ82790.1], *O. mykiss* Nkaα1b [AAQ82789.1], *O. mykiss* Nkaα1c [AAQ82788.1], *O. mossambicus* Nkaα1 [AAD11455.2], *O. mossambicus* Nkaα3 [AAF75108.1], *Salmo salar* Nkaα1 [ACN10460.1], *Sarotherodon melanotheron* Nkaα1 [ADB03120.1], *Trematomus bernacchii* Nkaα3 [AAY30258.1] and *Ciona intestinalis* Nkaα3 [XP_002124837.1] as an outgroup. Phylogenetic analysis was done using the neighbor-joining method with 100 bootstrap replicates using the Phylip package [38].

### Tissue Expression of *nkaα1*, *nkaα3a* and *nkaα3b*

Total RNA (1 µg) isolated from the brain, operculum membrane, liver, anterior gut, posterior gut, kidney and skin of *M. albus* kept in freshwater were reverse transcribed into cDNA using oligo(dT)_18_ primer and the RevertAid™ first strand cDNA synthesis kit (Fermentas International Inc.). PCR was performed on the cDNAs of these tissues using the specific qPCR primers (Table 1) to detect the mRNA expression of each gene in various tissues. Each PCR was carried out in 10 µl reaction volumes using Dreamtaq polymerase (Fermentas International Inc.) with thermal cycling conditions: 95°C for 3 min, followed by 30 cycles of 95°C for 30 s, 55°C for 30 s, 72°C for 30 s and a final extension of 72°C for 10 min. PCR products were then separated by electrophoresis in 2% agarose gel.

### Quantitative Real-time PCR (qPCR)

Total RNA (1 µg) from the brain samples of *M. albus* was reverse transcribed using random hexamer primers with RevertAid™ first strand cDNA synthesis kit (Fermentas International Inc.). qPCR was performed in triplicates using a StepOnePlus™ Real-Time PCR System (Life Technologies Corporation, Carlsbad, California). The mRNA expression of *nkaα1*, *nkaα3a* and *nkaα3b* in the brain of *M. albus* were determined using specific qPCR primers (Table 1). For *nkaα*, the specificity of each pair of qPCR primers was verified by PCR using three different plasmid clones containing fragments of *nkaα1*, *nkaα3a* and *nkaα3b* as templates. The identities of these plasmid clones had been verified through cloning and sequencing (see above). The specificity of each pair of primers was demonstrated by the presence of a single band using the plasmid clones of the targeted *nkaα* isoform as the template and the absence of detectable band using the plasmid clones of the other two isoforms.

Since it is essential to compare the mRNA expression of the three nkaα isoforms in the brain of *M. albus*, the method of absolute quantification with reference to a standard curve was adopted in this study. Relative quantitation methods produce only fold-change data, which do not allow the interpretation of which isoform being the predominant one being expressed in a certain condition. Although absolute quantification provides more information, it is considered to be more labor-intensive than relative quantification. Absolute quantification is not commonly adopted because of the necessity to create reliable standards for quantification and include these standards in every PCR. Therefore, to determine the absolute quantity of transcripts of each of the 3 *nkaα* in a qPCR reaction, efforts were made to produce a pure amplicon (standard) of a defined region of each of the 3 cDNA from the brain of *M. albus* following the method of Gerwick et al. [39]. The amplicon sizes were 137 bp, 115 bp and 122 bp for *nkaα1*, *nkaα3a* and *nkaα3b*, respectively. PCR was performed with a specific set of qPCR primers (Table 1) and cDNA as a template in a final volume of 25 µl with the following cycling conditions: initial denaturation 95°C for 3 min, followed by 35 cycles of 95°C for 30 s, 60°C for 30 s and 72°C for 30 s and 1 cycle of final extension of 72°C for 10 min. The PCR product was separated in a 2% agarose gel then excised and purified using FavorPrep™ Gel Purification Mini Kit (Favorgen Biotech Corp., Ping-Tung, Taiwan). The nucleotide fragment in the purified product was cloned using pGEM®-T Easy vector (Promega Corporation). The presence of the insert in the recombinant clones was confirmed by sequencing. The cloned circular plasmid was quantified using a spectrophotometer with a Hellma TrayCell.

The standard cDNA (template) was serially diluted (from 10^6^ to 10^2^ specific copies per 2 µl). The PCR reactions contained 5 µl of 2× Fast SYBR® Green Master Mix (Life Technologies Corporation), 0.2 pmol l^−1^ of forward and reverse primers each (Table 1) and 1 ng of sample cDNA or various quantities of standard in a total volume of 10 µl. Cycling conditions were 95°C for 20 s (1 cycle), followed by 40 cycles of 95°C for 3 s and 60°C for 30 s. Data (Ct values) were collected at each elongation step. A melt curve analysis was performed after each run by increasing the temperature from 60°C to 95°C in 0.3°C increments to confirm the presence of only a single product. The PCR products obtained were also separated in a 2% agarose gel to verify the presence of a single band. A standard curve was obtained from plotting threshold cycle (Ct) on the Y-axis and the natural log of concentration (copies µl^−1^) on the X-axis. The Ct slope, PCR efficiency, Y-intercept and correlation coefficient (*R^2^*) were calculated using the default setting of StepOne™ Software v2.1 (Life Technologies Corporation). Diluted standards were stored at −20°C. The amplification efficiencies for *nkaα1*, *nkaα3a* and *nkaα3b* were 91.2%, 97.1% and 83.8%, respectively. The quantity of transcript in a sample was determined from the linear regression line derived from the standard curve and expressed as copy number per ng cDNA. Copy numbers were calculated from the Ct values of the standards.

### Western Blot

Brain samples of *M. albus* were homogenized three times in five volumes (v/w) of ice cold buffer containing 50 mmol l^−1^ Tris HCl, (pH 7.4), 1 mmol l^−1^ EDTA, 150 mmol l^−1^ NaCl, 1 mmol l^−1^ NaF, 1 mmol l^−1^ Na_3_VO_4_, 1% NP-40, 1% sodium deoxycholate, 1 mmol l^−1^ PMSF, and 1×HALT protease inhibitor cocktail (Pierce, Rockford, USA) at 24,000 rpm for 20 s each with 10 s intervals using the Polytron PT 1300D homogenizer (Kinematica, Luzern, Switzerland). The homogenate was centrifuged at 10,000×g for 20 min at 4°C. The protein concentration in the supernatant obtained was determined according to the method of Bradford [35] and adjusted to 2 µg µl^−1^ with Laemmli buffer [40]. Samples were heated at 70°C for 15 min, and then kept at −80°C until analysis.

Proteins were separated by SDS-PAGE (8% acrylamide for resolving gel, 4% acrylamide for stacking gel) under conditions as described by Laemmli [40] using a vertical mini-slab apparatus (Bio-Rad Laboratories). Proteins were then electrophoretically transferred onto PVDF membranes using a transfer apparatus (Bio-Rad Laboratories). After transfer, membranes were blocked with 10% skim milk in TTBS (0.05% Tween 20 in Tris-buffered saline: 20 mmol l^−1^ Tris-HCl; 500 mmol l^−1^ NaCl, pH 7.6) for 1 h before being incubated overnight at 4°C with NKAα3 specific anti-NKA antibody (1∶800 dilution; Y-13, Santa Cruz Biotechnology Inc., Texas, USA) or NKAα5 antibody (1∶800 dilution; Developmental Studies Hybridoma Bank/DSHB, Iowa City, IA, USA) or pan-actin antibody (1∶5000 dilution; Thermo Fisher Scientific, United Kingdom). The anti-NKAα5 antibody was developed by Douglas M. Farmbrough (Johns Hopkins University, MD, USA) and is known to react pan-specifically with Nka α-subunit isoforms in fish and other animals. All primary antibodies were diluted in 1% bovine serum albumin in TTBS. The membranes were then incubated in horseradish peroxidase-conjugated secondary antibodies (anti-goat for NKAα3; 1∶40,000 dilution; goat anti-mouse for NKAα5 and pan-actin; 1∶10,000; Santa Cruz Biotechnology Inc.) for 1 h at room temperature. Bands were visualized by chemiluminescence (Western Lightning™, PerkinElmer Life Sciences, Boston, MA, USA) using X-ray film (Thermo Fisher Scientific) and were processed by a Kodak X-Omat 3000 RA processor (Kodak, Rochester, NY, USA). The films were scanned using CanonScan 4400F flat bed scanner in TIFF format at 300 dpi resolution. Densitometric quantification of band intensities were performed using ImageJ (version 1.40, NIH), calibrated with a 37 step reflection scanner scale (#R3705-1C; Stouffer Graphic Arts, South Bend, IN, USA). Results were presented as relative protein abundance of Nka normalized with actin.

### Statistical Analysis

Results were presented as means ± standard errors of the mean (S.E.M.). Differences between means were evaluated using one-way analysis of variance (ANOVA), followed by multiple comparisons of means by Tukey’s post-hoc test. Ratios were processed with arcsine transformation before statistical analysis. Differences were regarded as statistically significant at *P*<0.05.

## Results

### Ammonia Concentration in the Brain of *M. albus* exposed to Ammonia

After 1 day or 6 days of exposure to 50 mmol l^−1^ NH_4_Cl, the concentration of ammonia in the brain of *M. albus* (* N* = 5 for each group) increased significantly to 2.85±0.43 and 4.38±0.82 µmol g^−1^, respectively, as compared with that of the freshwater control (*N* = 5; 0.83±0.21 µmol g^−1^). No mortality of experimental fish was recorded.

### Nucleotide Sequences of *nkaα* and Phylogenetic Analysis of the Deduced Nkaα Amino Acid Sequences

Three different *nka α*-subunit isoforms were cloned and sequenced from the brain of *M. albus*. The complete cDNA coding sequence of *nkaα1* [GenBank: KC620448] consisted of 3078 bp, coding for 1025 amino acids, with an estimated molecular mass of 113 kDa. Similarly, the full length of *nkaα3a* [GenBank: KC620449] cDNA sequence was comparable to that of *nkaα1* with 3069 bp and coded for 1022 amino acids, with an estimated molecular mass of 113 kDa. However, the full cDNA coding sequence of *nkaα3b* [GenBank: KC620450] was longer at the 3′ end, containing 3282 bp which translated into 1093 amino acids, with a calculated molecular mass of 120 kDa.

**Figure 1:**
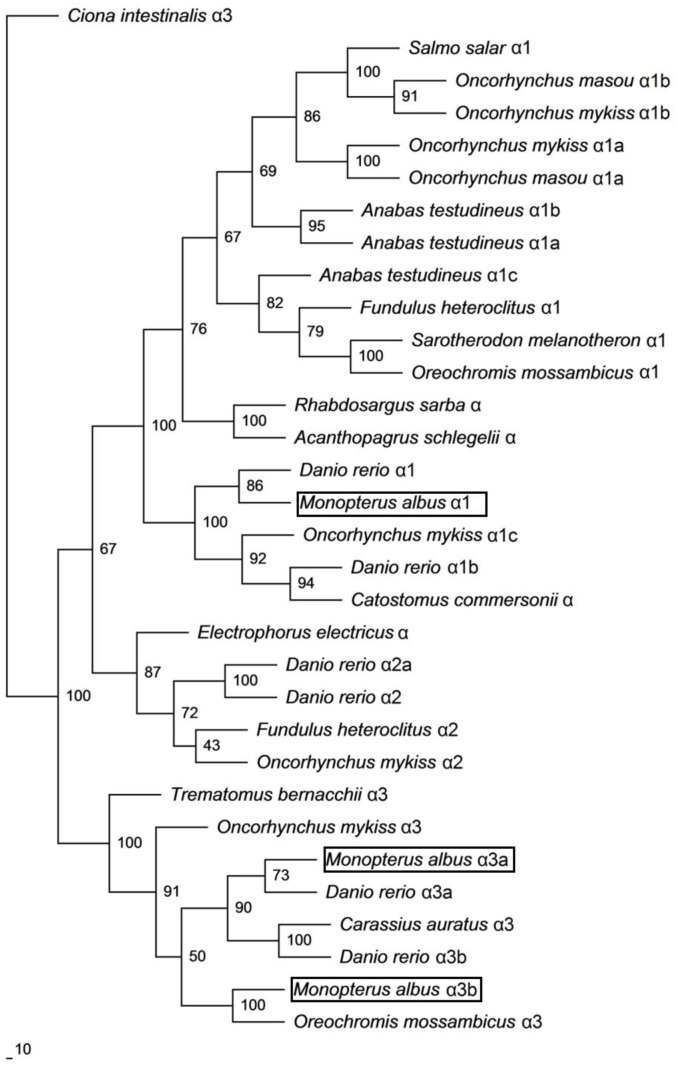
Phylogenetic analysis of Na^+^/K^+^-ATPase (Nka) α1, Nkaα3a and Nkaα3b. A phylogenetic tree to illustrate the relationship between Nkaα1, Nkaα3a and Nkaα3b from the brain of *Monopterus albus* and Nka of selected teleost species. Numbers presented at each branch point represent bootstrap values from 100 replicates. *Ciona intestinalis* Nka is used as an outgroup.

A phylogenetic analysis confirmed that Nkaα1 of *M. albus* was grouped together with teleost Nkaα1 and is distinct from various Nkaα2 and Nkaα3 isoforms (Fig. 1). On the other hand, both Nkaα3a and Nkaα3b of *M. albus* were closely related to teleost Nkaα3 instead of Nkaα1 and Nkaα2 isoforms (Fig. 1).

**Figure 2:**
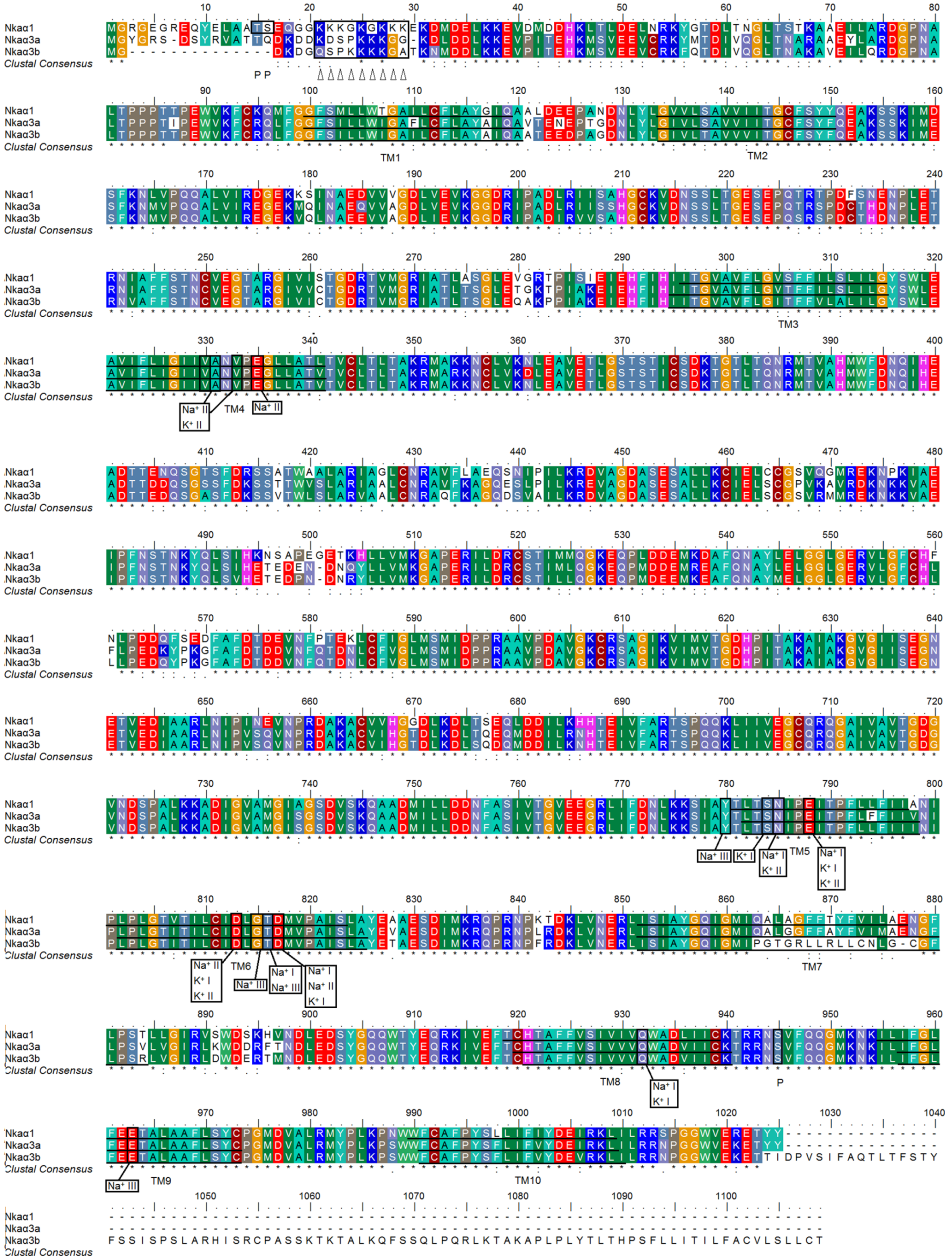
Molecular characterization of Na^+^/K^+^-ATPase (Nka) α1, Nkaα3a and Nkaα3b. A multiple amino acid sequence alignment of Nkaα1, Nkaα3a and Nkaα3b from the brain of *Monopterus albus*. Identical amino acid residues are indicated by asterisks, strongly similar amino acids are indicated by colons and weakly similar amino acids are indicated by periods. The ten predicted transmembrane regions (TM1–TM10) are underlined. Vertical boxes represent coordinating residues for Na^+^ or K^+^ binding. ‘P’ denotes phosphorylation sites and triangles indicate the lysine-rich region. The transmembrane domains of Nkaα1, Nkaα3a and Nkaα3b of *M. albus* were predicted using MEMSATS and MEMSAT-SVA provided by PSIPRED protein structure prediction server.

The deduced amino acid sequences of Nkaα1, Nkaα3a and Nkaα3b from the brain of *M. albus* had ten predicted transmembrane domains and an alignment of these three deduced amino acid sequences, together with those of *O. mossambicus*, *X. laevis*, *R. norvegicus* and *H. sapiens*, revealed large areas of conserved regions (Fig. 2; Fig. S1). Based on the homology modeling of human NKA α-subunit [41], three Na^+^ and two K^+^ binding sites are known to be present in the NKA α-subunit. Indeed, the coordinating residues responsible for Na^+^ or K^+^ binding were found to be highly conserved across all three Nka α-subunit isoforms present in the brain of *M. albus* (Fig. 2). A region containing a lysine-rich sequence that plays a critical role in cation binding and occlusion [42] was also present in all three isoforms of the Nka α-subunits with Nkaα1 containing the greatest number of lysine residues. In addition, potential phosphorylation sites that could serve as targets for protein kinase A [43] were present in all three Nka α-subunit isoforms. While potential targets for regulatory phosphorylation by protein kinase C [43] were found to be present in Nkaα1, they were absent from Nkaα3a and Nkaα3b (Fig. 2).

### Comparison of Nka α-subunits from the Brain of *M. albus* with those from the Gills of *Anabas testudineus*

**Figure 3:**
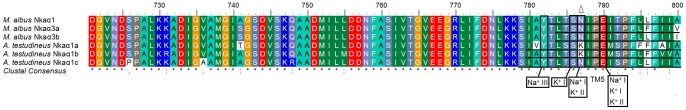
Analysis of Na^+^/K^+^ binding sites of Na^+^/K^+^-ATPase (Nka) α1, Nkaα3a and Nkaα3b. A multiple amino acid sequence alignment of a region of Nkaα1, Nkaα3a and Nkaα3b from the brain of *Monopterus albus*, with Nkaα1a [GenBank: JN180940], Nkaα1b [GenBank: JN180941] and Nkaα1c [GenBank: JN180942] from the gills of *Anabas testudineus*. Identical amino acid residues are indicated by asterisks, strongly similar amino acids are indicated by colons and weakly similar amino acids are indicated by periods. Vertical boxes represent coordinating residues for Na^+^ or K^+^ binding. A triangle indicates the amino acid residue that is identical in Nkaα1c but different in Nkaα1a and Nkaα1b.

An alignment of the amino acid sequences of Nkaα1, Nkaα3a and Nkaα3b from the brain of *M. albus*, with Nkaα1a, Nkaα1b and Nkaα1c from the gills of *A. testudineus* [44] revealed that all of them shared the highest percentage similarity with Nkaα1c (Table 2). More importantly, a detailed analysis of the amino acid residues which constitute one of the K^+^-binding sites revealed that those of Nkaα1, Nkaα3a and Nkaα3b from the brain of *M. albus* were identical to Nkaα1c but distinct from those of Nkaα1a and Nkaα1b from the gills of *A. testudineus* (Fig. 3).

**Table 2:** Percentage similarity between Na^+^/K^+^-ATPase (Nka) α-subunits from the brain of *Monopterus albus* and those from gills of *Anabas testudineus*.

*M. albus*	*A. testudineus*	Similarity
Nkaα1 [KC620448]	Nkaα1c [JN180942]	90.1%
	Nkaα1b [JN180941]	85.2%
	Nkaα1a [JN180940]	80.2%
Nkaα3a [KC620449]	Nkaα1c [JN180942]	82%
	Nkaα1b [JN180941]	79.3%
	Nkaα1a [JN180940]	76.4%
Nkaα3b [KC620450]	Nkaα1c [JN180942]	74.6%
	Nkaα1b [JN180941]	70.6%
	Nkaα1a [JN180940]	68.5%

The percentage similarity between the deduced amino acid sequence of Nkaα1, Nkaα3a and Nkaα3b from the brain of *M. albus* with Nkaα1a, Nkaα1b and Nkaα1c from the gills of *A. testudineus* obtained from GenBank (accession numbers in brackets; Ip et al. [44]). Sequences are arranged in a descending order of similarity.

### Tissue Expression of the Three *nka α*-subunit Isoforms

**Figure 4:**
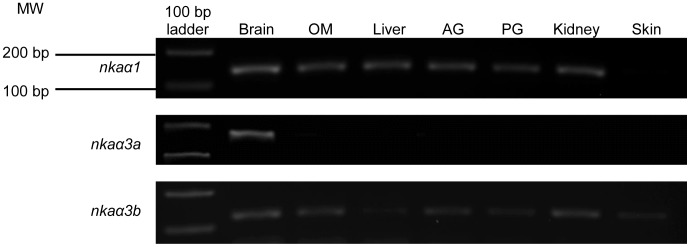
Tissue expression of *Na^+^/K^+^-ATPase* (*nka*) *α1*, *nka*α*3a* and *nkaα3b*. mRNA expression of *nkaα1*, *nkaα3a* and *nkaα3b* in the brain, operculum membrane (OM), liver, anterior gut (AG), posterior gut (PG), kidney and skin of *Monopterus albus* kept in freshwater.

*nkaα1* was expressed in the brain, operculum membrane, liver, anterior gut, posterior gut and kidney, but not the skin, of *M. albus* kept in freshwater (Fig. 4). By contrast, *nkaα3a* was expressed only in the brain. On the other hand, *nkaα3b* was detected readily in the brain, operculum membrane, kidney and anterior gut, but weakly in the operculum membrane, posterior gut and skin (Fig. 4).

### Effects of Exposure to Environmental Ammonia on the mRNA Expression of the Three *nka α*-subunit Isoforms in the Brain of *M. albus*

**Figure 5:**
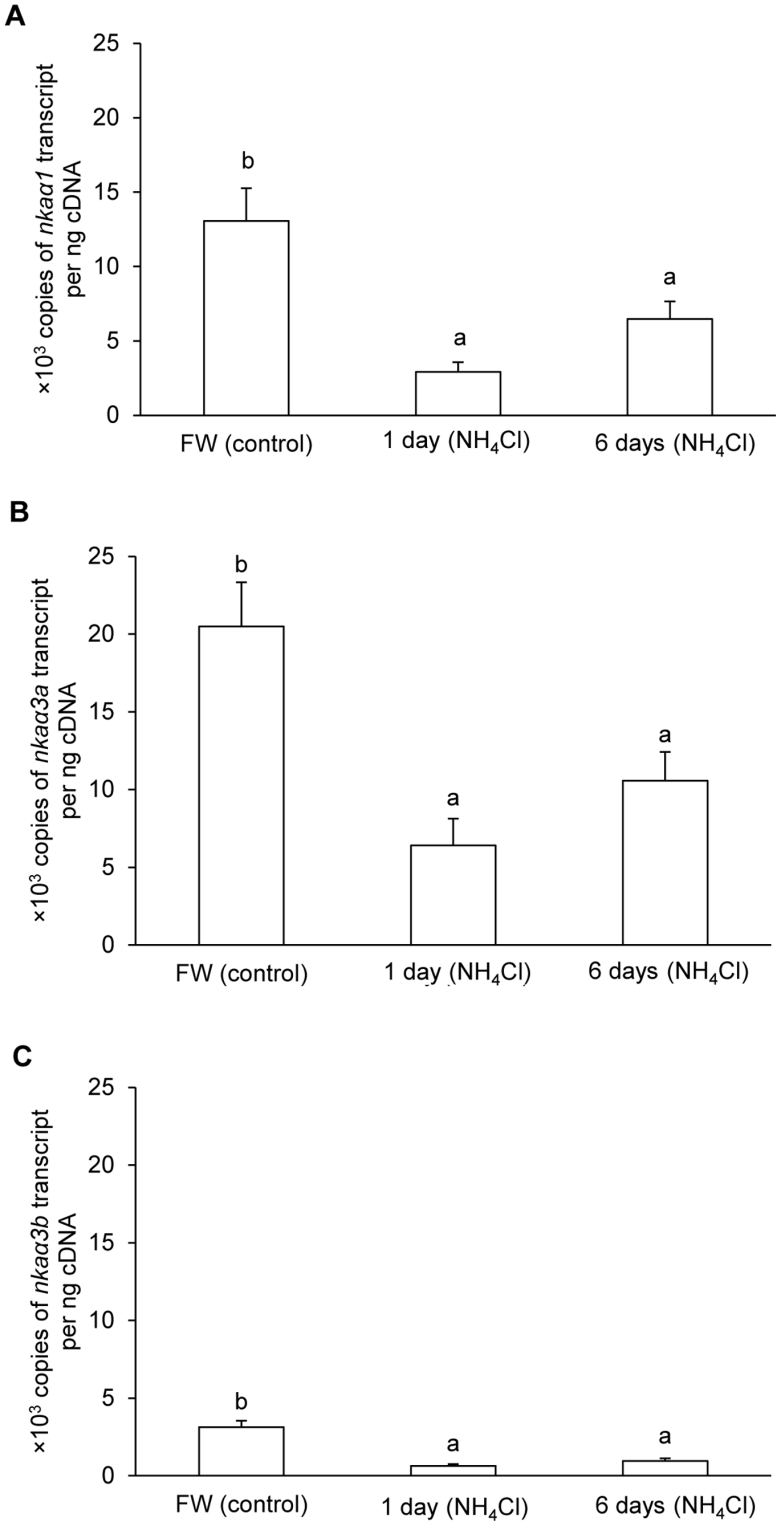
Effects of ammonia exposure on *Na^+^/K^+^-ATPase* (*nka*) *α1*, *nka*α*3a* and *nkaα3b* mRNA expression. Absolute quantification (copies of transcript per ng cDNA) of mRNA expression of (A) *nkaα1*, (B) *nkaα3a* and (C) *nkaα3b*, in the brain of *Monopterus albus* kept in freshwater (FW; control), or after exposure to 50 mmol l^−1^ NH_4_Cl for 1 day or 6 days. Results represent mean+S.E.M. (*N* = 5). Means not sharing the same letter are significantly different (*P*<0.05).

In the brain of *M. albus* kept in freshwater, the quantity (copies per ng cDNA) of *nkaα3a* was the highest (∼20,000 copies), followed by *nkaα1* (∼13,000 copies) and *nkaα3b* (∼3,000 copies). Ammonia exposure led to significant decreases in the mRNA expression of all three *nka α*-subunit isoforms in the brain of *M. albus*. After 1 and 6 days of exposure to 50 mmol l^−1^ NH_4_Cl, the mRNA expression of *nkaα1* decreased by 77.7% and 50.4%, respectively (Fig. 5A). The corresponding decreases in mRNA expression were 68.7% and 48.4% for *nkaα3a* (Fig. 5B) and 79.4% and 69.3% for *nkaα3b* (Fig. 5C).

### Overall Protein Abundance of Nka α-subunit during Environmental Ammonia Exposure

**Figure 6:**
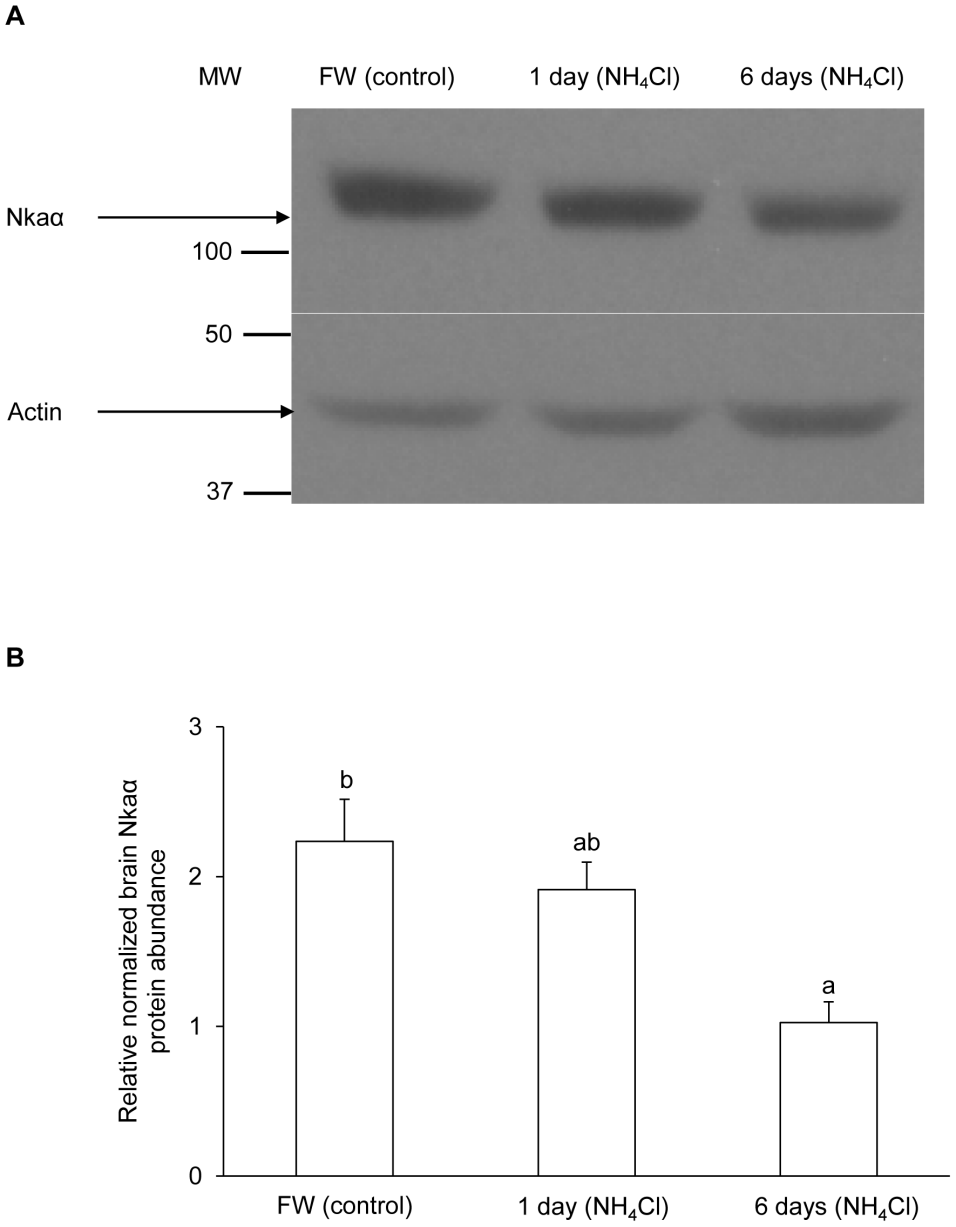
Effects of ammonia exposure on total Na^+^/K^+^-ATPase (Nka) protein abundance. Protein abundance of Nka, based on the α5 anti-NKA antibody which is known to react with all Nka/NKA α-isoforms, in the brain of *Monopterus albus* kept in freshwater (FW; control) or exposed to 50 mmol l^−1^ NH_4_Cl for 1 day or 6 days. (A) An example of the immunoblots of Nka and actin. (B) The intensity of the Nka band normalized with respect to that of actin. Results represent mean+S.E.M. (*N* = 3). Means not sharing the same letter are significantly different (*P*<0.05).

**Figure 7:**
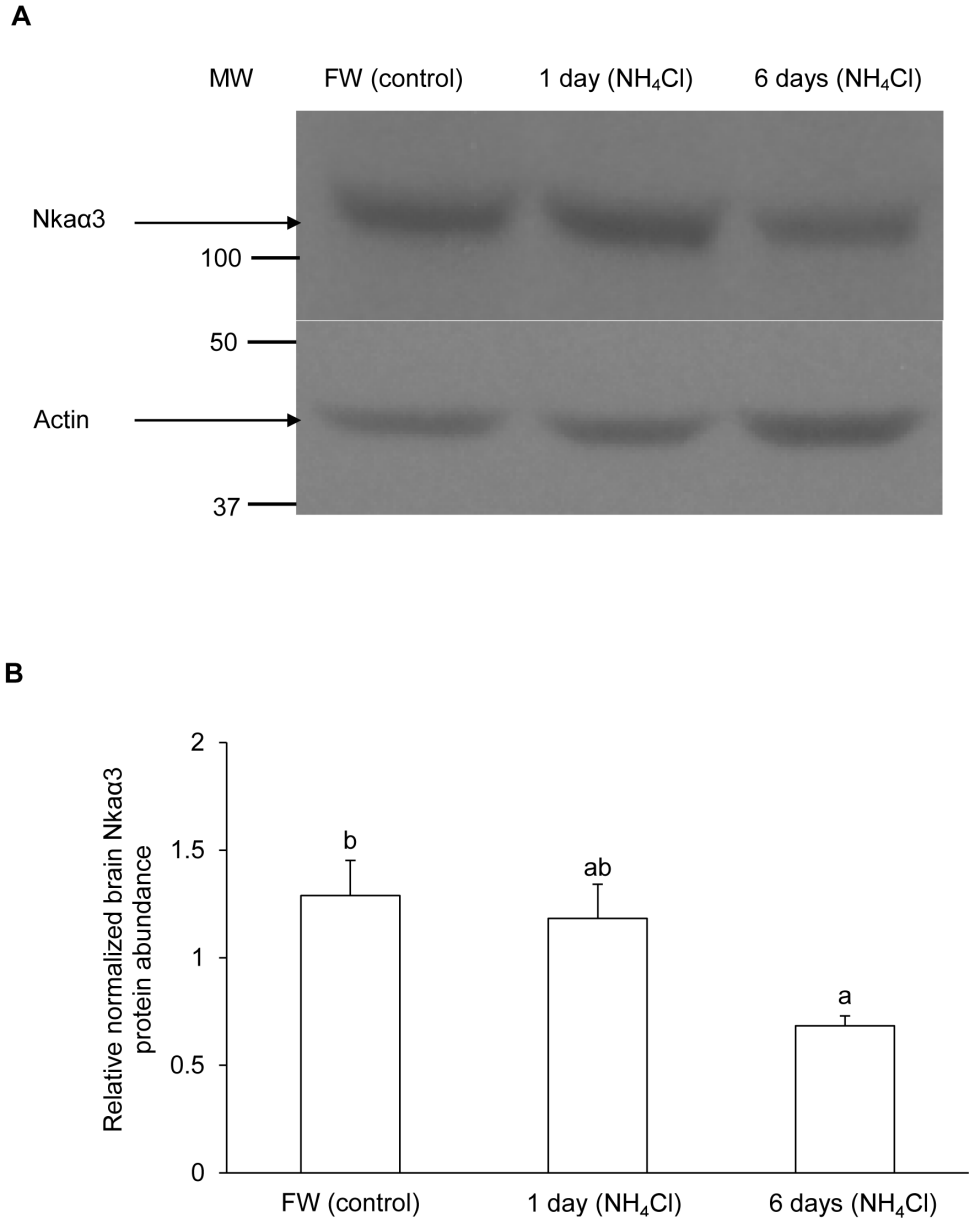
Effects of ammonia exposure on Na^+^/K^+^-ATPase α3 (Nkaα3) protein abundance. Protein abundance of Nkaα3, based on anti-NKAα3 antibody, in the brain of *Monopterus albus* kept in freshwater (FW; control) or exposed to 50 mmol l^−1^ NH_4_Cl for 1 day or 6 days. (A) An example of the immunoblots of Nkaα3 and actin. (B) The intensity of the Nka band normalized with respect to that of actin. Results represent mean+S.E.M. (*N* = 3). Means not sharing the same letter are significantly different (*P*<0.05).

The protein abundance of Nka α-subunit (Fig. 6) from the brain of *M. albus*, based on the α5 anti-NKA antibody which is pan-specific for Nka α-subunit isoforms, and that of Nkaα3 (Fig. 7), based on α3-specific antibody, decreased significantly after exposure to 50 mmol l^−1^ of NH_4_Cl for 6 days as compared with the freshwater control.

### Effectiveness of NH_4_^+^, Substituting for K^+^, to Activate Nka/NKA from the Brains of *M. albus* and *M. musculus*

**Figure 8:**
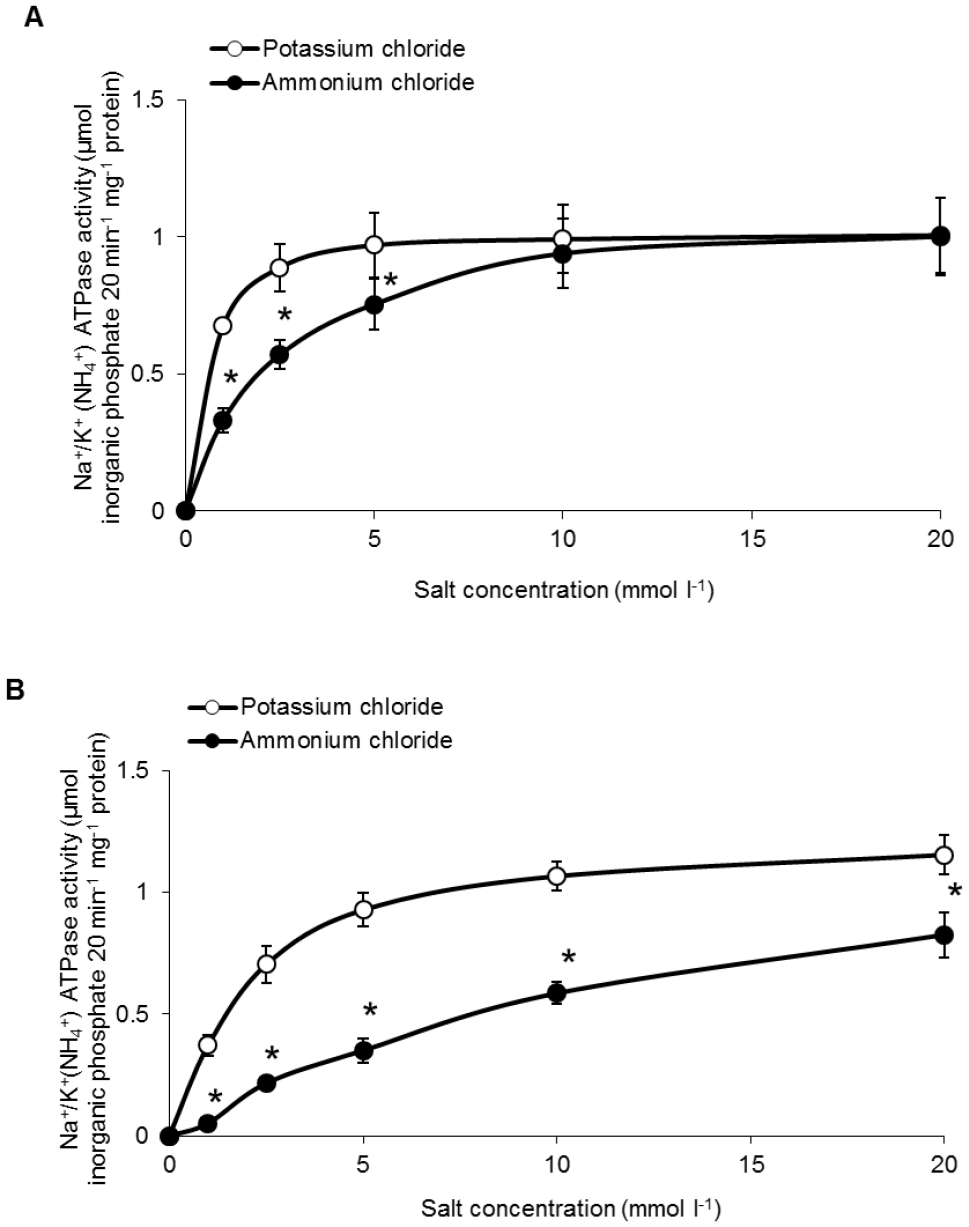
Effects of varying K^+^ or NH_4_^+^ concentrations on Na^+^/K^+^-ATPase or Na^+^/NH_4_^+^-ATPase activities from brains of *Mus musculus* and *Monopterus albus*. Specific activity (µmol inorganic phosphate 20 min^−1 ^mg^−1^ protein) of NKA were determined from the brain of (A) *M. musculus* and (B) *M. albus* kept in freshwater with varying concentrations of K^+^ or NH_4_^+^. Results represent mean ± S.E.M. (*N* = 5). Asterisks indicate significant difference from the corresponding potassium-induced specific activity (*P*<0.05).

The Na^+^/NH_4_^+^-ATPase activities from the brain of *M. musculus* assayed at low NH_4_Cl concentrations (1, 2.5 or 5 mmol l^−1^) were significantly lower than the NKA activities assayed at corresponding KCl concentrations (Fig. 8A). However, at high concentrations of KCl or NH_4_Cl (10 or 20 mmol l^−1^), there was no significant difference between the NKA and the Na^+^/NH_4_^+^-ATPase activities. These results confirm that NH_4_^+^ could effectively substitute for K^+^ as a substrate for NKA from the mouse at high substrate concentrations. In contrast, the brain Na^+^/NH_4_^+^-ATPase activities of *M. albus* (Fig. 8B) assayed at 1, 2.5, 5, 10 or 20 mmol l^−1^ NH_4_Cl were significantly lower than the Nka activities assayed at similar KCl concentrations (Fig. 8B).

At substrate concentrations of 1, 2.5, 5, 10 and 20 mmol l^−1^, the effectiveness of NH_4_^+^ substituting for K^+^ to activate Nka (expressed as ratios of Na^+^/NH_4_^+^-ATPase to Na^+^/K^+^-ATPase activities) from the brain of *M. albus* were 0.16, 0.33, 0.43, 0.60 and 0.71, respectively (Table 3). In comparison, the effectiveness of NH_4_^+^ substituting for K^+^ to activate NKA from the brain of *M. musculus* were significantly higher (0.55, 0.74, 0.78, 0.94 and 0.99, respectively) than those of *M. albus* (Table 3). Exposure to 50 mmol l^−1^ NH_4_Cl for 6 days had no significant effect on the effectiveness of NH_4_^+^ to activate Nka activity from the brain of *M. albus* (Table 3).

**Table 3:** A comparison of Na^+^/K^+^-ATPase and Na^+^/NH_4_^+^-ATPase activities, from the brains of *Monopterus albus* or *Mus musculus*, at various concentrations of K^+^/NH_4_^+^.

KCl or NH_4_Cl concentration (mmol l^−1^)	Ratio of Na^+^/NH_4_^+^ATPase activity to Na^+^/K^+^ATPase activity		
	*M. musculus*	*M. albus*	
		Freshwater	50 mmol l^−1^ NH_4_Cl for 6 days
1	0.55±0.04^b^	0.16±0.03^a^	0.26±0.04^a^
2.5	0.74±0.08^b^	0.33±0.05^a^	0.33±0.02^a^
5	0.78±0.03^b^	0.43±0.05^a^	0.49±0.03^a^
10	0.94±0.04^b^	0.60±0.04^a^	0.70±0.04^a^
20	0.99±0.06^b^	0.71±0.08^a^	0.85±0.03^ab^

Effectiveness of NH_4_^+^ substituting for K^+^ (expressed as ratio of Na^+^/NH_4_^+^-ATPase activity to Na^+^/K^+^-ATPase activity) to induce Nka activities from the brain of *M. musculus*, or from the brain of *M. albus* kept in freshwater or exposed to 50 mmol l^−1^ NH_4_Cl for 6 days.

Values are means ± S.E.M. (*N* = 5).

Means not sharing the same letter are significantly different, *P*<0.05.

## Discussion

Air-breathing fishes, particularly amphibious ones, are equipped with various strategies to ameliorate ammonia toxicity during emersion or ammonia exposure [12]–[14]. Active ammonia excretion, exhibited by *P. schlosseri* [45], [46] and *A. testudineus* [47], is theoretically the most effective strategy to maintain low internal (plasma and tissue) ammonia concentrations. Recent reports on *A. testudineus* have revealed that both active salt excretion during seawater acclimation and active NH_4_^+^ excretion during ammonia exposure (in freshwater) involve similar transport mechanisms (cystic fibrosis transmembrane conductance regulator, Nkcc1 and Nka) but two different types of Nka-immunoreactive cells in its gills [44], [48], [49]. In comparison, *M. albus* has degenerate gills and is incapable of active ammonia excretion. Therefore, it is imperative for *M. albus* to develop high tolerance of ammonia at the cellular level, especially in the brain. Our results suggested for the first time a possible relationship between the high brain ammonia tolerance of *M. albus* and (1) the high effectiveness of its brain Nka to differentiate K^+^ from NH_4_^+^, and (2) the ability of its brain to down-regulate the mRNA and protein expression of *nkaα*/Nkaα when confronted with ammonia toxicity.

### The Brain of *M. Albus* Expressed *nkaα1*, *nkaα3a* and *nkaα3b*, but not *nkaα2*

Three *nka α*-subunit isoforms were expressed in the brain of *M. albus*. Based on phylogenetic analysis, they were identified as *nkaα1*, *nkaα3a* and *nkaα3b*. The PCR primers used in this study were designed against the highly conserved regions of *nkaα1*, *nkaα2* and *nkaα3*. However, cloning and sequencing results confirmed the expression of *nkaα1* and *nkaα3*, but not *nkaα2*, in the brain of *M. albus*. To further verify the lack of expression of *nkaα2* in the brain of *M. albus* in response to ammonia exposure, we performed suppression subtractive hybridization PCR using control brain as driver and brain of fish exposed to 50 mmol l^−1^ NH_4_Cl as tester, and confirmed the absence of *nkaα2* in the forward and reverse libraries (Y. K. Ip, unpublished results). In contrast, Semple et al. [50] reported the expression of *nkaα2* in the brain (and muscle) of *F. heteroclitus*, while Guynn et al. [51] reported the expression of Nkaα1, Nkaα2 and Nkaα3 in the brain of the Antarctic nototheniid, *Trematomus bernacchii*, and the temperate nototheniid, *Notothenia angustata*. Thus, the lack of expression of *nkaα2* in the brain of *M. albus* is uncommon, and could have a physiological reason.

In mammalian brain, three isoforms, *NKAα1*, *NKAα2* and *NKAα3*, have been identified [27], [32]. In adult mouse brain and in cultured mouse brain cells, the NKAα1 isoform is expressed in both neurons and astrocytes, while NKAα2 is an astrocyte specific isoform and NKAα3 is expressed in neurons [52]. These three NKA α-subunit isoforms differ with regard to their Na^+^, K^+^ and ATP sensitivity [53], [54], but they all have a specific binding site for ouabain and its analogues, the cardiotonic steroids [55], [56]. Rodent NKAα1 has a much lower affinity to ouabain (KD of 9.3 µM and 1.5 µM in cultured mouse astrocytes and neurons, respectively) than NKAα2 (KD of ∼80 nM in cultured mouse astrocytes) and NKAα3 (KD of ∼110 nM in cultured mouse neurons) and NKAα1 from other species [57].

Similar to the brain of *M. albus,* the spiral ganglion and organ of Corti of rat cochlea were found to express NKAα1 and NKAα3 but not NKAα2 [58]. It is probable that NKAα1 and NKAα3 may play more prominent roles in handling the physiological demands of myelinated axons. In comparison with NKAα1, NKAα3 has relatively low affinity for intracellular Na^+^ [52], [53], [59], high affinity for ATP [52], and a lack of inhibition at hyperpolarized potentials [59], [60]. Thus, NKAα3 appears to be especially suited for myelinated axons which can sustain high rates of activity leading to an increase in intracellular Na^+^ concentrations, a depletion of ATP, and prolonged hyperpolarization of membrane potentials [58]. Expression of various rat NKA α-subunit isoforms in *Xenopus* oocytes reveals that NKAα2 isoform has a three-fold higher sensitivity to extracellular Na^+^ when compared with NKAα1 and NKAα3, causing the NKAα2 isoform to be more sensitive to the membrane potential [61]. Hence, it is logical to deduce that the lack of expression of *nkaα2* in the brain of *M. albus* could have a physiological relevance related to its high brain ammonia tolerance.

Recently, Xue et al. [62] investigated (i) effects of ammonia on mRNA and protein expression of *N* *kaα1*/NKAα1 and *N* *kaα2*/NKAα2 in primary cultures of mouse astrocytes; (ii) effects of hyperammonia obtained by urease injection on mRNA and protein expression of *N* *kaα1*/NKAα1 and *N* *kaα2*/NKAα2 in the mouse brain *in* *vivo*; and (iii) effect on observed upregulation of gene expression of *N* *kaα2*/NKAα2 induced by tyrphostin AG1478, an inhibitor of the EGF receptor-tyrosine kinase, PP1, an inhibitor of Src, and GM6001, an inhibitor of Zn^2+^-dependent metalloproteinases in cultured mouse astrocytes. They [62] established that mRNA and protein expression of *N* *kaα2*/NKAα2, but not *N* *kaα1*/NKAα1, were upregulated in cultured astrocytes after 1–4 days of exposure to 3 or 5 mmol l^−1^ ammonia, and that similar upregulation, contrasted by a down-regulation of the neuronal *N* *kaα3*/NKAα3, occurred in the hyperammonemic brain. Based on the effects of the inhibitors (AG1478, PP1 and GM6001) and how they affect the mRNA and protein expression of *N* *kaα2*/NKAα2, Xue et al. [62] concluded that ammonia activated the formation of an endogenous ouabain-like compound, which binds to NKA and activates Src, which in turn stimulates the receptor-tyrosine kinase of the EGF receptor. This led to the activation of the Ras, Raf, MEK pathway and phosphorylation of ERK1/2, which eventually causes an upregulation of *N* *KAα2* mRNA expression. Assuming that a similar molecular pathway exists in fish brain in general, the lack of expression of *nkaα2* in the brain of *M. albus* could be an important adaptation to its mode of living (air-breathing and emersion) and conditions of its habitat (high environmental ammonia concentration), as it would contribute in part to its high brain ammonia tolerance.

### Molecular Characterization of Nkaα1, Nkaα3a and Nkaα3b from Brain of *M. albus*

Three Na^+^ and two K^+^ binding sites are known to be present in the NKA α-subunit [41], [63]. The coordinating residues present in the binding sites are arranged within the transmembrane domains such that the release of one type of cation coordinates with the binding of the other. Based on the homology modeling of human NKA α-subunit [41], these coordinating residues are found to be highly conserved in all three isoforms of the Nka α-subunit obtained from the brain of *M. albus*. Furthermore, it has been established that Na^+^ and K^+^ are occluded within NKA during each turnover of the pump and this occlusion requires conformational changes in the enzyme [64]. Proteolytic cleavage at a lysine-rich region near the N-terminal alters the equilibrium between the El and E2 conformations [65]. Hence, this conformational shift could involve the movement of the lysine-rich sequence, which could serve as a movable, ion-selective gate, controlling the passage of Na^+^ and K^+^ during certain stages of the transport process [42]. Indeed, the highly conserved lysine-rich sequence is present in Nkaα1, Nkaα3a and Nkaα3b from the brain of *M. albus*. Thus, this indirectly implies that the mechanisms of ion transport in Nka from *M. albus* could be similar to those of other species and they might share close structural-functional relationships.

Our results indicate that Nka activity could be regulated by phosphorylation in the brain of *M. albus*. Both cAMP-dependent protein kinase A and protein kinase C are known to be involved in the phosphorylation of the NKA α-subunit [66] although the functional effects of protein kinases remain controversial [67]. One possible site of cAMP-dependent protein kinase A phosphorylation, serine-945 [43], was present in all three Nka α-subunit isoforms from the brain of *M. albus*. However, out of these three Nka α-subunit isoforms, only Nkaα1 contains cAMP-dependent protein kinase C phosphorylation sites, serine-16 and threonine-15 [43]. Hence based on previous reports [43], [66], [67], it is probable that Nkaα3a and Nkaα3b could be regulated in a cAMP-dependent protein kinase A-dependent and cAMP-dependent protein kinase C-independent manner in the brain of *M. albus*, as in rat neostriatal cells [68].

Morth et al. [69] reported that there was a 26-fold reduction in Na^+^ affinity when five amino acid residues (delKETYY) were deleted from the C-terminal of NKA. In *M. albus*, the KETYY motif was present in both Nkaα1 and Nkaα3a, but it is missing from Nkaα3b, the C-terminus of which had 84 more amino acids. These results indicate that the Na^+^ affinity of Nkaα3b could be different from those of Nkaα1 and Nkaα3a.

### The Implications of High Similarity in K^+^ Binding sites between Nkaα1, Nkaα3a and Nkaα3b of *M. albus* and Nkaα1c of *A. testu*dineus

A detailed analysis of the amino acid residues constituting the K^+^ binding sites of Nkaα1, Nkaα3a and Nkaα3b from the brain of *M. albus* revealed that they are identical to those of Nkaα1c, but different from those of Nkaα1a and Nkaα1b, from the gills of *A. testudineus*. Exposure of *A. testudineus* to 100 mmol l^−1^ NH_4_Cl in freshwater resulted in a significant increase in the mRNA and protein expression of Nkcc1 in the gills [48]. Hence, it is probable that NH_4_^+^ enters mitochondrion-rich cells through basolateral Nkcc1 before being actively transported across the apical membrane. However, the operation of Nkcc1 during active ammonia excretion would lead to an increase in the intracellular Na^+^ concentration of the mitochondrion-rich cells. Therefore, an up-regulation of Nka activity would be necessary to remove the excess Na^+^. In order to maintain intracellular K^+^ homeostasis, the gills of *A. testudineus* must express more than one type of Nka α-isoform, with at least one isoform that can differentiate K^+^ from NH_4_^+^, rendering NH_4_^+^ ineffective to substitute for K^+^ to induce Nka activity. Indeed, Ip et al. [44] reported that three *nka α*-isoforms (*α1a*, *α1b* and *α1c*) were expressed in the gills of *A. testudineus*, and their results suggested that *nkaα1a* was a freshwater isoform while *nkaα1b* was a seawater isoform. They also demonstrated that environmental ammonia exposure led to significant increases in the mRNA expression of *nkaα1c*, the overall Nka protein abundance, the Nka activity, and the *K*_m_ for K^+^ and NH_4_^+^ in the gills of *A. testudineus*. Since the increase in *K*_m_ for NH_4_^+^ was much greater than that for K^+^, ammonia exposure apparently induced a decrease in the effectiveness of NH_4_^+^ to substitute for K^+^ in the activation of Nka, and the up-regulation of *nkaα1c* expression served to remove excess Na^+^ from, and to transport K^+^ in preference to NH_4_^+^ into, mitochondrion-rich cells in order to maintain intracellular Na^+^ and K^+^ homeostasis [44]. Therefore, the similarity in the K^+^ binding sites between all three Nka α-subunit isoforms from the brain of *M. albus* and Nkaα1c from the gills of *A. testudineus* indicate that the overall Nka activity from the brain of *M. albus* might exhibit high substrate specificity for K^+^.

### The Nka from the Brain of *M. albus* can Differentiate K^+^ from NH_4_^+^ Better than the NKA from the Brain of *M. musculus*

Indeed, our results reveal for the first time that the Nka from the brain of *M. albus* has a high specificity for K^+^, as compared to NH_4_^+^, at physiological concentrations (∼1 mmol l*^−^* ^1^) of K^+^ or NH_4_^+^. For control fish, NH_4_^+^ was only 15% effective in substituting for K^+^ to induce Nka activity. More importantly, our results confirm that Nka from the brain of *M. albus* had a greater K^+^ specificity than NKA from the mouse brain. This would imply that, when confronted with high brain ammonia concentration, cells in the brain of *M. albus* could maintain intracellular K^+^ homeostasis and low intracellular ammonia concentration through the normal functioning of Nka with only a low level of K^+^ being substituted by NH_4_^+^. Thus, the high K^+^ specificity of Nka from the brain of *M. albus* could have a major contribution to its extraordinarily high brain ammonia tolerance. As for the mouse brain, the ineffectiveness of its NKA to differentiate K^+^ from NH_4_^+^ could be one of the contributing factors to its low tolerance of ammonia [1]. Exposure of *M. albus* to ammonia had no significant effect on the effectiveness of NH_4_^+^ or K^+^ to activate Nka from its brain. Hence, a down-regulation of the mRNA and protein expression of these *nka*/Nka α-subunit isoforms would be essential to further ameliorate the deleterious effects of ammonia under hyperammonia conditions.

### Down-regulation of mRNA and Protein Expression of *nka*/Nka α-subunit Isoforms in the Brain of *M. albus* Exposed to Ammonia

Mammalian brains have low brain ammonia tolerance, and an increase in brain ammonia concentration is known to induce higher *NKA* gene expression and NKA activity. It has been established that an injection of large doses of ammonia into rats leads to the depletion of brain ATP [29]. Kosenko et al. [29] reported that an injection with ammonia into rat increased the brain NKA activity by 76%, which could be prevented by a previous injection of MK-801, an antagonist of the NMDA receptor. After normalizing NKA activity in samples from ammonia-injected rats by *in* *vitro* incubation with phorbol 12-myristate 13-acetate, an activator of protein kinase C, Kosenko et al. [29] obtained results indicating that ammonia-induced ATP depletion was mediated by the activation of NMDA receptor, which resulted in decreased protein kinase C-mediated phosphorylation of NKA and, therefore, increased NKA activity and increased consumption of ATP. It has also been reported that ammonia increases the production of ouabain-like substances and NKA activity in cultured mouse astrocytes [70]. Thus, increased activity of NKA could also be the result of enhanced production of ouabain-like compounds, as cultured rat astrocytes react to prolonged exposure to a high concentration of ouabain with an upregulation of NKAα1 [71].

By contrast, we report for the first time that 1 day or 6 days of exposure to ammonia resulted in significant decreases in mRNA expression of *nkaα1*, *nkaα3a* and *nkaα3b* in the brain of *M. albus*. In addition, there were significant decreases in the protein abundance of total Nka α-subunit isoforms (based on the commercially available pan-specific antibody α5), and Nkaα3 (based on the commercially available α3-specific antibody) in the brain of *M. albus* after 6 days of exposure to ammonia. Therefore, it is probable that reduction in the mRNA expression and protein abundance of *nka*/Nka α-subunit isoforms in the brain of *M. albus* exposed to ammonia could directly ameliorate the severity of ammonia toxicity, leading to high brain ammonia tolerance.

In mammals, swelling of astrocytes represents the most prominent neuropathological abnormality in acute liver failure [72], and ammonia has been shown to induce swelling of astrocytes *in* *vivo* [73], and *in* *vitro* [74]. Aquaporin 4, which acts as a water channel, has been implicated in the swelling process [75]. Recently, Illarionova et al. [76] reported that aquaporin 4 could assemble with its regulator metabotropic glutamate receptor 5 and NKA, forming a macromolecular transporting microdomain in astrocytes. Therefore, it is probable that the reduction in the mRNA expression and protein abundance of *nka*/Nka α-subunit isoforms in the brain of *M. albus* exposed to ammonia could suppress the function of aquaporin 4 and ameliorate the severity of ammonia-induced astrocyte swelling and brain edema, the confirmation of which awaits future studies.

### Perspective

Our results suggest for the first time that the ability to down-regulate the mRNA expression of *nkaα1*, *nkaα3a* and *nkaα3b* and protein abundance of Nka α-subunit isoforms in the brain could be some of the contributing factors to the extraordinarily high brain ammonia tolerance in *M. albus*. Another contributing factor could be the ineffectiveness of NH_4_^+^, as compared with K^+^, to activate Nka from the brain of *M. albus*. Efforts are being made in our laboratory to determine the localization of Nka in the brain of *M. albus*, and its functional relationship with other transporters, e.g. Nkcc1 and aquaporin. Since exposure to environmental ammonia also resulted in a reduction in *nkcc1b*/Nkcc1b expression [26], it is highly probable that these two transporters work in concert to control NH_4_^+^ influx into brain cells to ameliorate the toxic effects of high environmental ammonia exposure.

## Supporting Information

Figure S1 **Multiple amino acid sequence alignment of Na^+^/K^+^-ATPase (Nka) α-subunits.** A multiple amino acid sequence alignment of Nkaα1, Nkaα3a and Nkaα3b from the brain of *Monopterus albus* was performed with those of *Oreochromis mossambicus* Nkaα1 [GenBank: AAD11455.2], *Xenopus laevis* NKAα1 [GenBank: NP_001084064.1], *Rattus norvegicus* NKAα1 [GenBank: NP_036636.1], *Homo sapiens* NKAα1 [GenBank: NP_000692.2], *O. mossambicus* Nkaα3 [GenBank: AAF75108.1], *X. laevis* NKAα3 [GenBank: NP_001080440.1], *R. norvegicus* NKAα3 [GenBank: NP_036638.1], and *H. sapiens* NKAα3 [GenBank: NP_689509.1]. Identical amino acid residues are indicated by asterisks, strongly similar amino acids are indicated by colons and weakly similar amino acids are indicated by periods. The ten predicted transmembrane regions (TM1–TM10) are underlined. Vertical boxes represent coordinating residues for Na^+^ or K^+^ binding. ‘P’ denotes phosphorylation sites and triangles indicate the lysine-rich region. The transmembrane domains of Nkaα1, Nkaα3a and Nkaα3b of *M. albus* were predicted using MEMSATS and MEMSAT-SVA provided by PSIPRED protein structure prediction server.
(TIF)
