# Supplementary figures and images for: Properties and Expression of Na+/K+-ATPase α-Subunit Isoforms in the Brain of the Swamp Eel, Monopterus albus, Which Has Unusually High Brain Ammonia Tolerance
Source: PLoS One. 2013 Dec 31;8(12):e84298. doi: 10.1371/journal.pone.0084298 (PMC3877266; doi:10.1371/journal.pone.0084298)

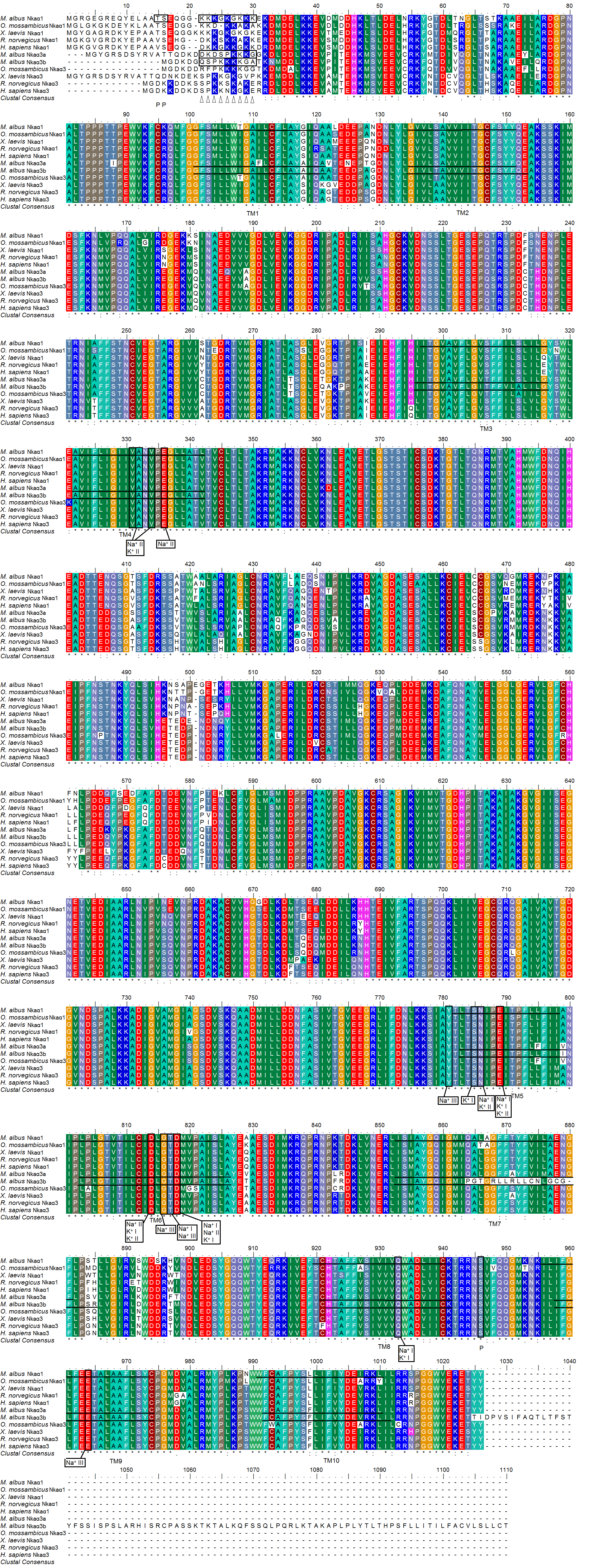

Supplement: Figure S1 — Multiple amino acid sequence alignment of Na+/K+-ATPase (Nka) α-subunits. A multiple amino acid sequence alignment of Nkaα1, Nkaα3a and Nkaα3b from the brain of Monopterus albus was performed with those of Oreochromis mossambicus Nkaα1 [GenBank: AAD11455.2], Xenopus laevis NKAα1 [GenBank: NP_001084064.1], Rattus norvegicus NKAα1 [GenBank: NP_036636.1], Homo sapiens NKAα1 [GenBank: NP_000692.2], O. mossambicus Nkaα3 [GenBank: AAF75108.1], X. laevis NKAα3 [GenBank: NP_001080440.1], R. norvegicus NKAα3 [GenBank: NP_036638.1], and H. sapiens NKAα3 [GenBank: NP_689509.1]. Identical amino acid residues are indicated by asterisks, strongly similar amino acids are indicated by colons and weakly similar amino acids are indicated by periods. The ten predicted transmembrane regions (TM1–TM10) are underlined. Vertical boxes represent coordinating residues for Na+ or K+ binding. ‘P’ denotes phosphorylation sites and triangles indicate the lysine-rich region. The transmembrane domains of Nkaα1, Nkaα3a and Nkaα3b of M. albus were predicted using MEMSATS and MEMSAT-SVA provided by PSIPRED protein structure prediction server. (TIF) [file pone.0084298.s001.tif]
